# Age effect on gray matter volume changes after sleep restriction

**DOI:** 10.1371/journal.pone.0228473

**Published:** 2020-02-06

**Authors:** Zhiliang Long, Fei Cheng, Xu Lei

**Affiliations:** Sleep and NeuroImaging Center, Faculty of Psychology, Southwest University, Chongqing, China; Xidian University, CHINA

## Abstract

Sleep deprivation disrupted functional and structural brain areas which are associated with cognition and emotion in healthy participants. However, the effect of age on the structural changes after sleep restriction remains unclear. In the current study, gray matter volume was calculated in 43 young adults and 37 old adults before and after sleep restriction. Two-way mixed analysis of variance (between-subject factor: deprivation; within-subject factor: age) was then employed to investigate differences in gray matter volume changes between young and old adults. Gaussian random field theory was applied for multiple comparison correction. Results revealed that sleep restriction decreases gray matter volume in the right thalamus, left precuneus, and postcentral gyrus. More importantly, we found a significant deprivation × age interaction effect mainly in the right dorsal/ventral anterior insula where the gray matter volume increased in young adults after sleep restriction but showed no difference in old adults. These findings highlight the crucial role of the anterior insula in the neural mechanisms underlying sleep lose, especially among young adults. The current work provided structural evidence for describing emotional dysfunction and suggests the potential effect of age on functional and structural changes after sleep restriction.

## Introduction

Sleep loss is a common and serious issue that exerts a negative effect on health. Insufficient sleep has been associated with impairment of cognition and emotion in healthy subjects [1]. The sleep–wake cycle has been observed to be disrupted in psychiatric and neurological disorders [2]. Thus, elucidating the mechanisms underlying sleep restriction is an important goal in basic and clinical neuroscience. Evidence indicates that aging has an effect on sleep. Participants with different age cohorts have been suggested to have different sensitivities to sleep lose [3].

It has been reported that better self-reported sleep is generally associated with better health outcomes, especially for mental health [4]. Sleep deprivation makes young brain resemble the old [5]. While old adults better tolerate sleep lose than young adults [3]. A study of psychomotor vigilance task found that after 40 hours sleep deprivation, the performance was declined more pronouncedly in the young than the old [6]. In addition, insufficient sleep might result in bad cognitive performance, in which the decrements were greater in the young as compared to the old [7]. A functional magnetic resonance imaging (MRI) study of verbal encoding task further reported that longer sleep time is involved in higher activation of anterior parahippocampus in old adults but low activation of the brain area in young adults, suggesting different mechanisms for maintaining cognitive performance [8]. Functional MRI studies also observed age effect on functional connectivity changes after sleep deprivation. For example, only young adults showed reduced resting-state functional connectivity of anterior insula after sleep restriction [9], and positive correlation between functional connectivity of medial temporal lobe network with sleep quality [10].

Sleep lose is associated with brain structural abnormalities. An animal study revealed that chronic sleep deprivation may negatively affect formation and maintenance of myelin [11]. A human study also suggested that acute sleep deprivation resulted in brain atrophy of distributed brain areas, which would be restored after one night sleep [12]. In healthy participants, sleep deprivation decreases thalamic gray matter volume [13], cortical thickness of medial parietal cortices [14], and prefrontal cortex [15,16]. Furthermore, the cortical atrophy was negatively correlated with sleepiness, suggesting structural plasticity after sleep deprivation [17]. Additionally, studies of sleep disorders also observed altered gray matter volumes of brain areas in the default mode network and the salience network [18,19].

Insufficient sleep was also involved in disrupted emotional processing [20]. For example, individuals with sleep deprivation had impaired dopaminergic reward functioning [21,22]. In addition, sleep-deprived participants had difficulty to discriminate between stimuli of different emotions. For instance, sleep-deprived individuals rate neutral images as more emotionally negative [23], and can not accurately identify threatening from affiliative facial signals [24]. A neuroimaging study revealed that anterior insula and anterior cingulate cortices play crucial role in impaired discrimination of negative emotions after one night of sleep deprivation [24].

Based on previous findings, the current study aims to explore the age effect on gray matter volume changes in individuals with sleep restriction. We hypothesize that (1) sleep restriction alters the gray matter volume of brain areas involved in emotional processing and (2) individuals within different age cohorts show different patterns of gray matter volume change.

## Materials and methods

### Study design and participants

The dataset was obtained from the Sleepy Brain Project (https://openneuro.org/datasets/ds000201). A total of 86 participants were included and classified into the young adult group (age, 20–30 years) and the old adult group (age, 65–75 years). This study features a cross-over comparison between one night sleep restriction (3 hours sleep) and full sleep. Participants were randomized to undergo both conditions in a counterbalanced order with an interval of approximately 1 month. In the interest of ecological validity, the participants slept in their own homes in both conditions. Participants were asked to fill in sleep diaries from 3 nights before the experiment, and told to avoid coffee and alcohol on the experimental day. On the night before imaging, sleep was monitored using ambulatory polysomnography. In the sleep restriction condition, the participants were instructed to go to bed 3 hours before the time they would usually get up and then get up at their normal time. MRI imaging was performed in the evening following sleep restriction or normal sleep. Before scanning, the participants were asked to complete several questionnaires, such as the positive and negative effect schedule (PANAS), Epworth sleepiness scale, insomnia severity index, and Karolinska Sleep Questionnaire such as normal time in bed, sleep quality index and snoring symptom index. PANAS scores were again recorded once after sleep restriction of all participants. Five subjects were excluded because their T1 images before or after sleep restriction were missing. One subject was excluded due to artifacts. Finally, 80 participants remained (37 old adults, 43 young adults). Other details can be found in previous studies [25,26]. The project was preregistered at clinicaltrials.gov (https://clinicaltrials.gov/ct2/show/NCT02000076), and this study was approved by the Regional Ethics Review Board of Stockholm (2012/1870–32). All participants provided written informed consent before participating in this work, and all experiments were performed in accordance with the Declaration of Helsinki and applicable local regulations. Details of the participants are provided in Table 1.

**Table 1:** Participant characteristics and sleep measures.

	Young (n = 43)	Old (n = 37)	p-value
Sex (female/male)	21/22	20/17	0.64^a^
ESS (mean±SD)	7.23±3.01	8.65±4.8	0.11^b^
ISI (mean±SD)	3.49±2.07	2.11±1.63	**0.002**^b^
KSQ sleep quality index (mean±SD)	5.26±0.43	5.19±0.48	0.52^b^
KSQ snoring symtom index (mean±SD)	5.88±0.32	5.65±0.54	**0.02**^b^

^a^Chi-square test.

^b^Two-tailed two sample t-test.

Bold p-values indicates significant difference in sleep measures between young group and old group. SD, standard deviation; ESS, Epworth Sleepiness Scale; ISI, Insomnia Severity Index; KSQ, Karolinska Sleep Questionnaire.

### Scan acquisition

The MRI data of the Sleepy Brain Project were acquired using a General Electric Discovery 3T MRI scanner. T1 structural images were scanned using a sagittal BRAVO sequence, a 24 cm field of view, and a 1 mm slice thickness. Other parameters used can be referenced from previous studies [25,26].

### Voxel-based morphometry analysis

All images were visually inspected for artifacts or structural abnormalities. Then, voxel-based morphometry (VBM) analysis was applied to the MRI images by using SPM12 (Wellcome Trust Centre for Neuroimaging, Institute of Neurology, UCL, London, UK; http://www.fil.ion.uncl.ac.uk/spm). Briefly, the origin of all structural images was manually set to the anterior commissure, after which the images were segmented into gray matter, white matter, and cerebrospinal fluid [27]. The segments were iteratively registered using the Diffeomorphic Anatomical Registration Through Exponentiated Lie algebra (DARTEL) toolbox [28], to produce a template of a group of individuals. The gray matter images were then spatially warped into the standard Montreal Neurological Institute space using affine spatial normalization and modulated by multiplication with the Jacobian determinant of the warp field [28]. Finally, the images were smoothed with an 8 mm full-width at half maximum (FWHM) isotropic Gaussian kernel.

Two-way mixed analysis of variance (between-subject factor: age, two levels; within-subject factor: deprivation, two levels) was employed to test differences between groups in terms of gray matter volume changes after sleep restriction. The individual total intracranial volume was included as covariance to remove the effect of different brain size. A Gaussian random field with p < 0.05 (voxel p < 0.01, Z > 2.3) was used for multiple comparison correction of the main effect of age, the main effect of deprivation, and the interaction effect of age × deprivation. Once a significant deprivation-related effect was observed, post-hoc analysis was employed for those brain areas showing significant effect. For the main effect of sleep restriction, we further investigated whether sleep restriction increases or decreases gray matter volume using independent two sample t-test. For the age × deprivation interaction effect, we further tested the difference in gray matter volume among young adults with and without sleep restriction, and old adults with and without sleep restriction using independent two sample t-test or paired t-test. Multiple comparisons were corrected using Bonferroni method with p<0.05/6. Notably, regions of interest (ROIs) for post-hoc analysis were selected using the “pick cluster” function in XJView (http://www.alivelearn.net/xjview8/).

Pearson correlation analysis was conducted to determine correlations between changes (post-deprivation vs. pre-deprivation) in the gray matter volume of the ROIs and changes (post-deprivation vs. pre-deprivation) in PANAS positive and negative scores in young adult and old adult separately. P values < 0.05 were considered statistically significant.

## Results

### Demographic results

Young and old participants had normal time in bed of 8.48±0.79 ours and 8.45±0.78 hours respectively. Analysis of ambulatory polysomnography data revealed that total sleep time in sleep restriction condition was 2.92±0.61 hours for young group, and 2.49±0.36 hours for old group. In the normal sleep condition, the total sleep time was 6.77±1.35 hours and 5.73±1.41 hours for young and old groups respectively. A mixed ANOVA was employed to test difference in PANAS scores. For positive PANAS score, we observed significant interaction effect (*F*_(1,77)_ = 9.73,*P* = 0.003), main effect of age (*F*_(1,77)_ = 18.58,*P* = 0.001), and main effect of deprivation (*F*_(1,77)_ = 22.1,*P* = 0.001). Post-hoc analysis indicated that sleep restriction decreases positive PANAS score only in young adult. For negative PANAS score, we only found significant main effect of age (*F*_(1,77)_ = 9,*P* = 0.004) with higher score in young adults. Old adults revealed a significant decrease in insomnia severity index and Karolinska Sleep Questionnaire snoring symptom index compared with young adults (Table 1).

### VBM results

**Fig 1:**
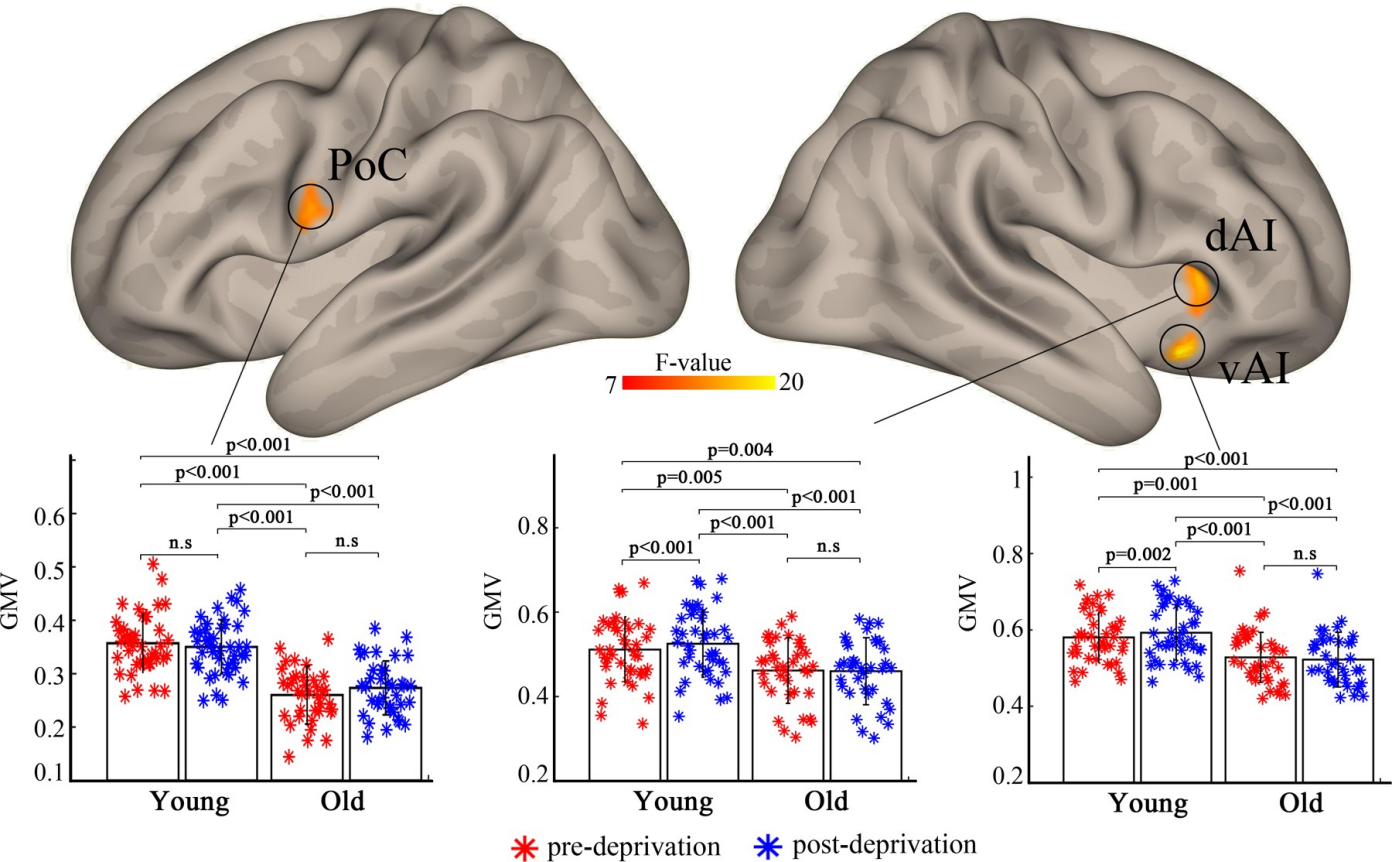
Significant age × deprivation interaction effect on gray matter volume in the left postcentral gyrus and right dorsal/ventral anterior insula. Post-hoc analysis revealed a larger volume of gray matter after sleep restriction in young adults but no difference in older adults. PoC, postcentral gyrus; dAI, dorsal anterior insula; vAI, ventral anterior insula.

**Fig 2:**
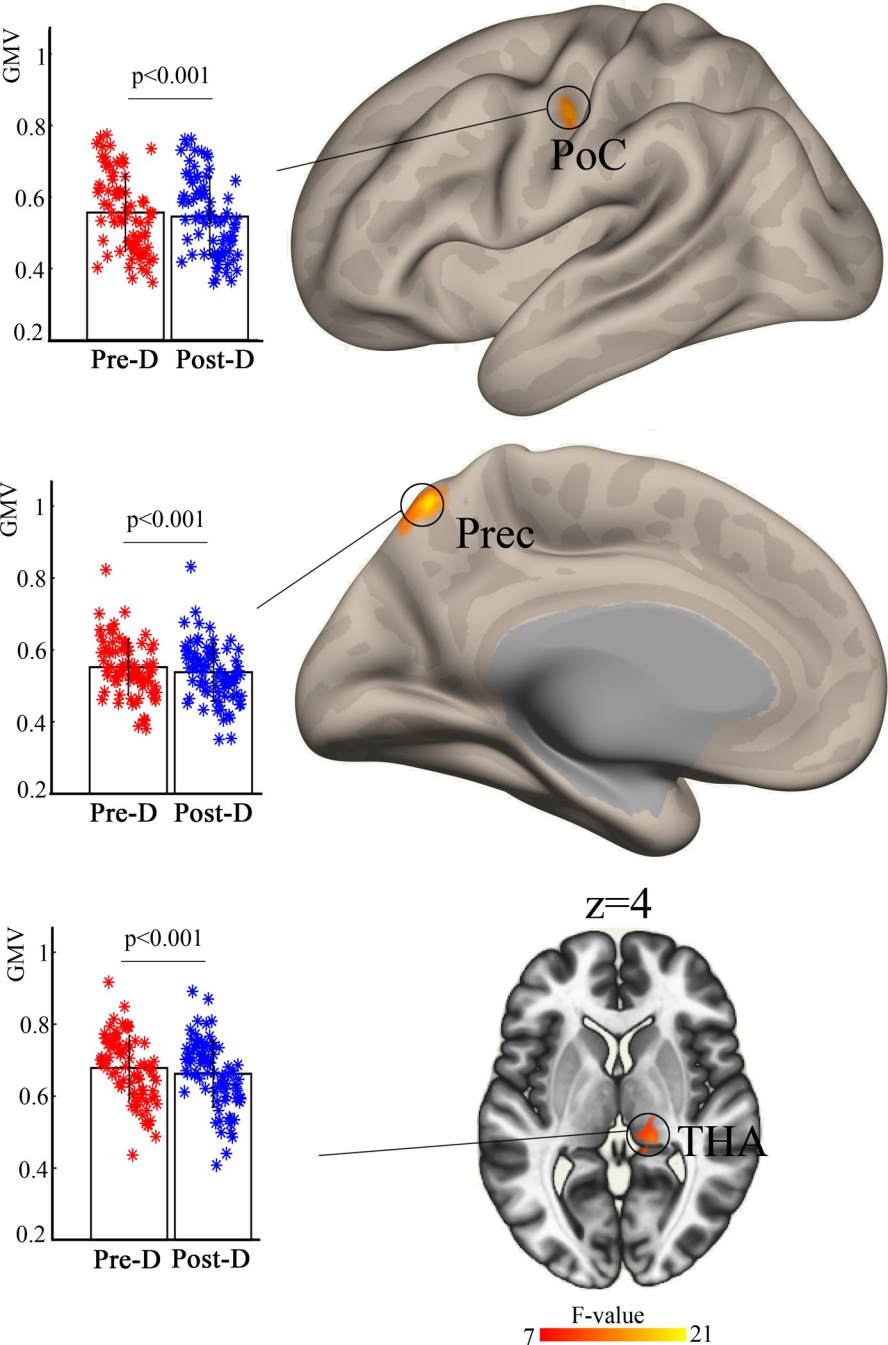
Significant main effect of deprivation on the left postcentral and precuneus and right thalamus. Post-hoc analysis indicated reduced gray matter volume in these brain areas. post-D, post-deprivation; pre-D, pre-deprivation; PoC, postcentral gyrus; Prec, precuneus; THA, thalamus.

Analysis of variance showed a significant age × deprivation interaction effect on the left postcentral gyrus (*F*_(1,77)_ = 11.1, partial *η*^2^ = 0.127), right dorsal anterior insula (dAI) (*F*_(1,77)_ = 11.8, partial *η*^2^ = 0.113), and ventral anterior insula (vAI) (*F*_(1,77)_ = 19.8, partial *η*^2^ = 0.159). Post-hoc analysis revealed increased gray matter volume in the right dAI and vAI after sleep restriction in young individuals but not in old individuals (Fig 1). A significant main effect of deprivation was observed in the left postcentral gyrus (*F*_(1,77)_ = 16.1, partial *η*^2^ = 0.171), left precuneus (*F*_(1,77)_ = 10.6, partial *η*^2^ = 0.173), and right thalamus (*F*_(1,77)_ = 20.8, partial *η*^2^ = 0.159) (Fig 2). Post-hoc analysis showed that these three brain areas exhibit decreased gray matter volume after sleep restriction.

Finally, we observed a significant main effect of age, that is, most brain areas of old adult exhibit decreased gray matter volume in comparison with those of young adults (S1 Fig). We did not find significant correlation (p>0.05) between gray matter volume change of those ROIs and positive or negative PANAS scores both in young and old groups.

## Discussion

In the present study, we observed a significant interaction effect between deprivation and age on the right anterior insula and a main effect of deprivation on the left postcentral gyrus and precuneus and right thalamus. These results provide structural evidence for the functional dysfunction of brain networks and suggest an age effect on morphological changes after sleep restriction.

We firstly observed a significant deprivation × age interaction effect on the anterior insula. Post-hoc analysis revealed that the gray matter volume of the anterior insula increases in the young cohort after sleep restriction but showed no change in the old cohort, suggesting a pathological mechanism underlying the increase in gray matter volume. The anterior insula, a key brain area of the salience network, is involved in attention and interoceptive and affective processes [29]. Neuroimaging evidence suggests that sleep deprivation can over-generalize the responsivity of the affective salience networks [30], which means participants with sleep deprivation cannot discriminate between stimuli of different emotional strengths. For example, deprivation individuals usually rate neutral images as more emotionally negative than their non-deprivation counterparts [23,24]. In addition, sleep deprivation alters the brain anticipation of cued emotional experience via the elevated activation of the anterior insula and anterior cingulate cortex [31]. A resting study revealed altered spontaneous neural activity of the insula of participants after sleep deprivation [32]. The enhanced gray matter volume of the anterior insula observed in the present study may suggest disrupted emotion discrimination and expression after sleep restriction. However, the gray matter volume of this area in old adults was preserved after sleep restriction. A possible interpretation is that old adults are more tolerant of sleep deprivation than young adults, which means the former are less affected by sleep restriction [6,33]. The decreased insomnia severity index in old adults compared with that in young adults observed in the current study provides direct evidence of this supposition. Another interpretation is that old adults may have less cortical plasticity than young adults. For example, a study of training task reported that motor training resulted in greater improvements in corticomotor excitability in young subjects compared with old subjects, suggesting reduced corticomotor plasticity with aging [34].

The significant main effect of deprivation was observed in the left precuneus, right thalamus, and left postcentral gyrus. These areas showed decreased gray matter volume after sleep restriction. Sleep deprivation has been suggested to impair short-term memory, which is associated with the reduced deactivation of the precuneus in participants with sleep deprivation [35]. Visual memory and novelty processing tasks demonstrate that sleep deprivation decreases the activation of the precuneus and ventral stream regions when processing object location [36,37]. Moreover, this decrease is correlated with poor recognition performance. The precuneus belongs to the default mode network. A resting study revealed that the spontaneous activity of the default mode network decreases after long-term total sleep deprivation [38]. Functional connectivity analyses further report increased functional connectivity between the precuneus and cerebellum [39] and amygdala [40]. The cortical thickness of the precuneus has been reported to decrease after 24 hours of sleep deprivation [14]. The decrease in gray matter volume observed in our study is consistent with previous results and suggests the dysfunction of the default mode network after sleep restriction.

The thalamus is believed to be a pivotal gating hub that integrates the flow of information from the periphery to the cortex [41]. Neuroimaging studies have demonstrated that sleep deprivation alters the thalamic activity during sustained attention. However, the profile of activity change in the thalamus is inconsistent. Some studies report increased activity under sleep loss conditions [42,43], while others observe diminished thalamic activity [44,45]. These inconsistencies may be due to differences in performance and cortical arousal provided by thalamic activity. The thalamus shows specific functional and structural connectivity with different cortices [46]. A resting study demonstrated that sleep deprivation decreases the thalamic functional connectivity with the cortex, mainly in the frontal and temporal cortex [47]. A morphological study reported reduced thalamic gray matter volume after long-term sleep deprivation [13], which is consistent with the results of the current study. The decreased gray matter volume of the postcentral gyrus after sleep restriction found in the present study is in accordance with that of a previous resting state study reporting altered activity and functional connectivity in this area [32,48].

Several limitations must be addressed. First, another independent datasets were needed to validate the findings observed in the current study. Second, the negative results (i.e., no difference in gray matter volume) found in old adults may be due to short-term sleep deprivation. Future studies must investigate the age effect on gray matter volume changes by applying long-term sleep deprivation. Third, this study only employed structural imaging data for analysis; thus, a relationship between brain function and structure under sleep restriction could not be found.

In conclusion, the current study investigated the effect of age on changes in gray matter volume after sleep restriction. We found a significant age × deprivation interaction effect on the anterior insula that suggests the dysfunction of this brain area in emotion discrimination and expression after sleep restriction. Moreover, sleep restriction decreased the gray matter volume of the default mode network and thalamus. This study provides a structural basis for the functional disturbance of the default mode network and salience network in sleep restriction individuals and highlights the role of age in sleep deprivation studies.

## Supporting information

S1 Fig Significant main effect of age showing decreased gray matter volume in nearly the entire cortical brain and subcortical cortex. (TIF)
