# Correction: Age effect on gray matter volume changes after sleep restriction

**DOI:** 10.1371/journal.pone.0246799

**Published:** 2021-02-04

**Authors:** Zhiliang Long, Fei Cheng, Xu Lei

In the Funding statement, the grant number from the funder Fundamental Research Funds for the Central Universities is listed incorrectly. The correct grant number is: SWU118004.

The ISI score values in Table 1 are incorrect. The authors have provided a corrected version here.

**Table 1:** Participant characteristics and sleep measures.

	Young (n = 43)	Old (n = 37)	p-value
Gender (female/male)	21/22	20/17	0.64^a^
ESS (mean±SD)	7.23±3.01	8.65±4.8	0.11^b^
ISI (mean±SD)	10.5±2.13	9±1.53	0.0014^b^
KSQ sleep quality index (mean±SD)	5.26±0.43	5.19±0.48	0.52^b^
KSQ snoring symtom index (mean±SD)	5.88±0.32	5.65±0.54	0.02^b^

^a^Chi-square test

^b^Two-tailed two sample t-test

Bold p-values indicates significant difference in sleep measures between young group and old group. SD, standard deviation; ESS, Epworth Sleepiness Scale; ISI, Insomnia Severity Index; KSQ, Karolinska Sleep Questionnaire.
