# Supplementary figures and images for: Age effect on gray matter volume changes after sleep restriction
Source: PLoS One. 2020 Feb 6;15(2):e0228473. doi: 10.1371/journal.pone.0228473 (PMC7004551; doi:10.1371/journal.pone.0228473)

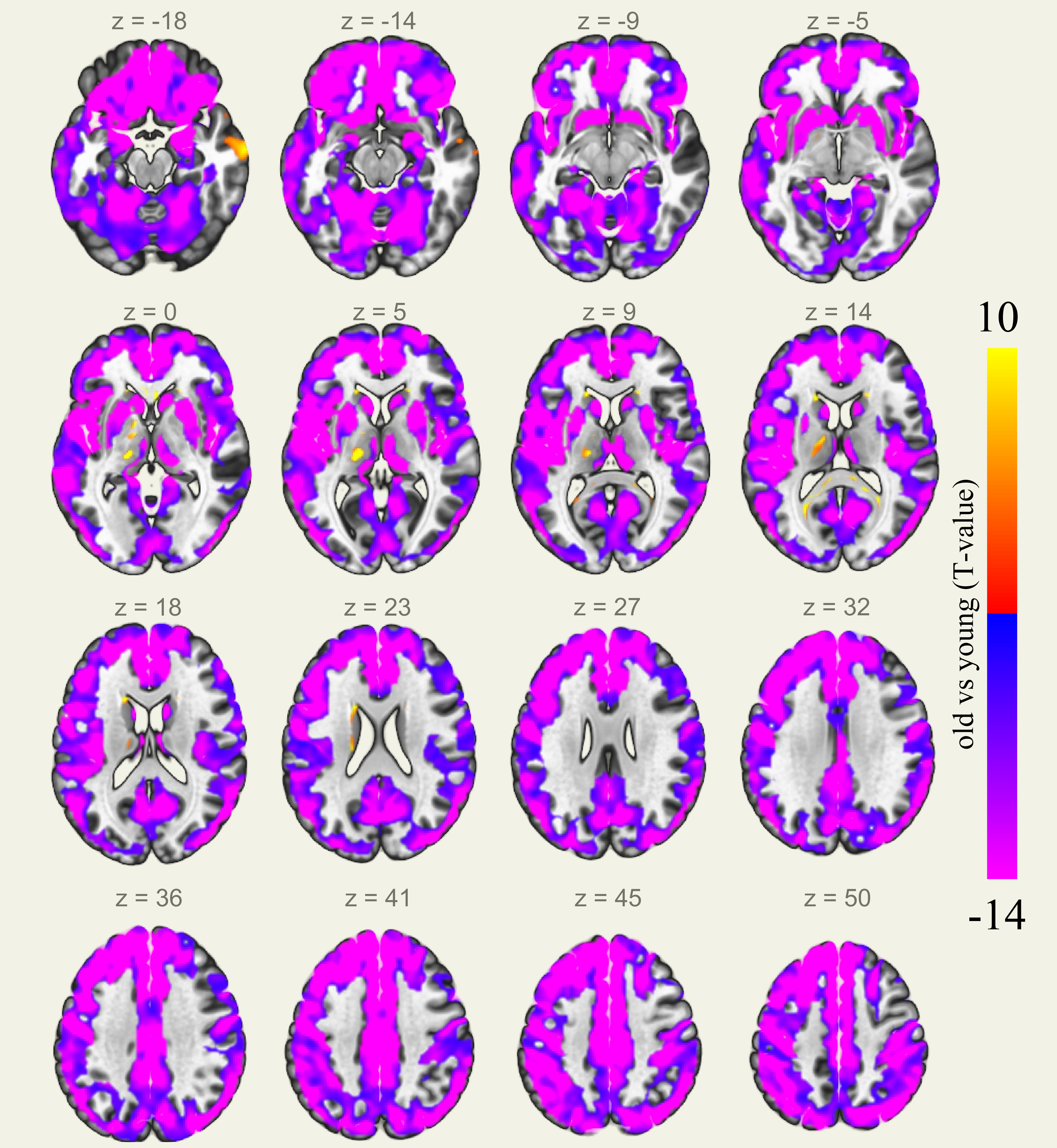

Supplement: S1 Fig — (TIF) [file pone.0228473.s001.tif]
